# Supplementary material for: Hemoglobin in the blood acts as a chemosensory signal via the mouse vomeronasal system
Source: Nat Commun. 2022 Feb 3;13:556. doi: 10.1038/s41467-022-28118-w (PMC8814178; doi:10.1038/s41467-022-28118-w)
Supplement: Supplementary file 1 — Supplementary Information [file 41467_2022_28118_MOESM1_ESM.pdf]

Supplementary Information

Hemoglobin in the blood acts as a chemosensory signal via the mouse vomeronasal system

Osakada et al.

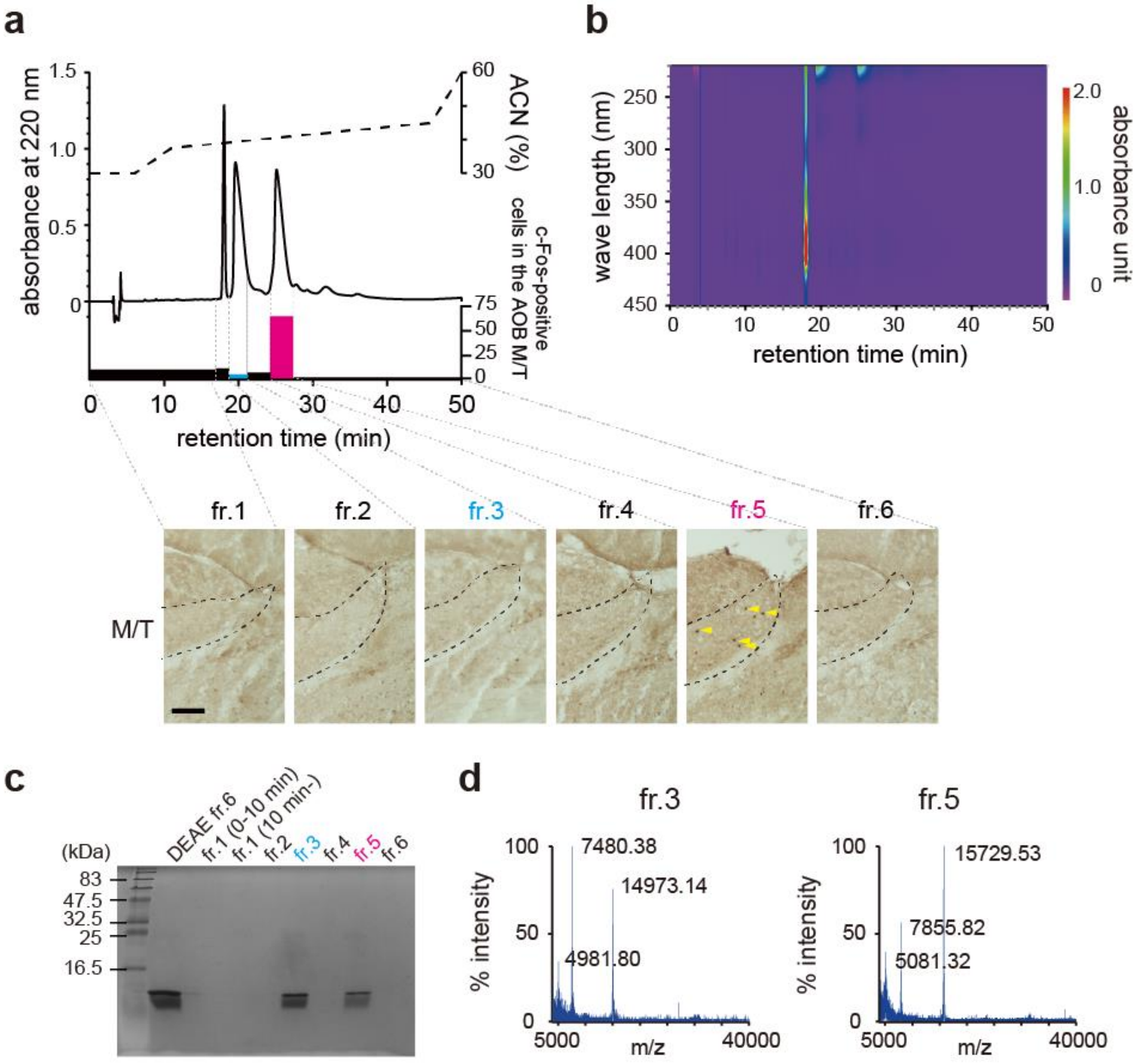

Supplementary Fig. 1 Additional information regarding the identification of the vomeronasal active molecule in C57BL/6 mouse blood**. a** Chromatogram of HPLC purification with a C4 column (also shown in Fig. 1g, top) and representative immunohistochemical images of the AOB sections from mice stimulated with each fraction (bottom). Arrowheads represent c-Fos-positive cells in the mitral/tufted cell layer (M/T) of the AOB. Scale bar, 100 µm. Each fraction was presented to one C57BL/6 male mouse respectively. The quantification of c-Fos-positive cells was with 30 sections from the both sides of the AOB. **b** Absorption spectrum of the chromatogram from the C4 column. Absorption at around 400 nm represents heme. **c** SDS-PAGE image with each sample fraction related to Fig. 1f and Supplementary Fig. 1a. **d** Mass spectra of fraction 3 and 5 from HPLC

purification with C4 columns. Fraction 3 exhibited a 14,973 Da main peak (left: α-globin) and fraction 5 exhibited a 15,729 Da peak (right: β-globin).

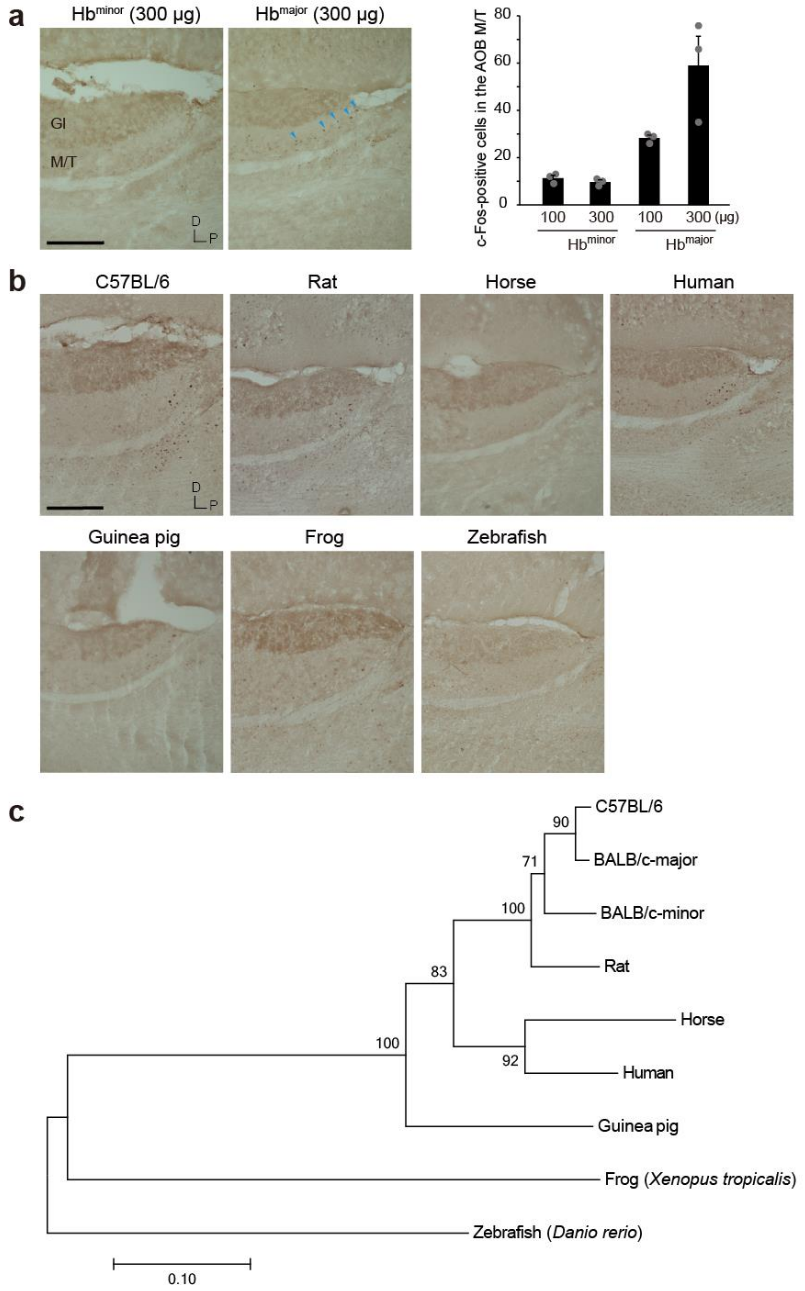

Supplementary Fig. 2 Representative images of AOB sections and a phylogenic tree of amino acid sequences of vertebrate hemoglobin**. a** Representative immunohistochemical images (left) and the number of total c-Fos-positive cells in the mitral/tufted cell layer (M/T) of the AOB sections from hemoglobin (Hb)-stimulated C57BL/6 male mice (right). $n = 3$. Error bars, S.E.M. Scale bar, 100 µm. **b** Representative immunohistochemical images of total c-Fos-positive cells in the mitral/tufted cell layer (M/T) of the AOB sections from C57BL/6

male mice stimulated with various vertebrate blood samples. Scale bar, 100 µm. *n* = 6 for C57BL/6, Rat, Horse, Human, Guinea pig, Frog, and Zebrafish, *n* = 8 for BALB/c-major, and *n* = 9 for BALB/c-minor. **c** Neighbor-joining phylogenic tree, constructed from the amino acid sequences of vertebrate hemoglobin using MEGA7[Ref.1,2].

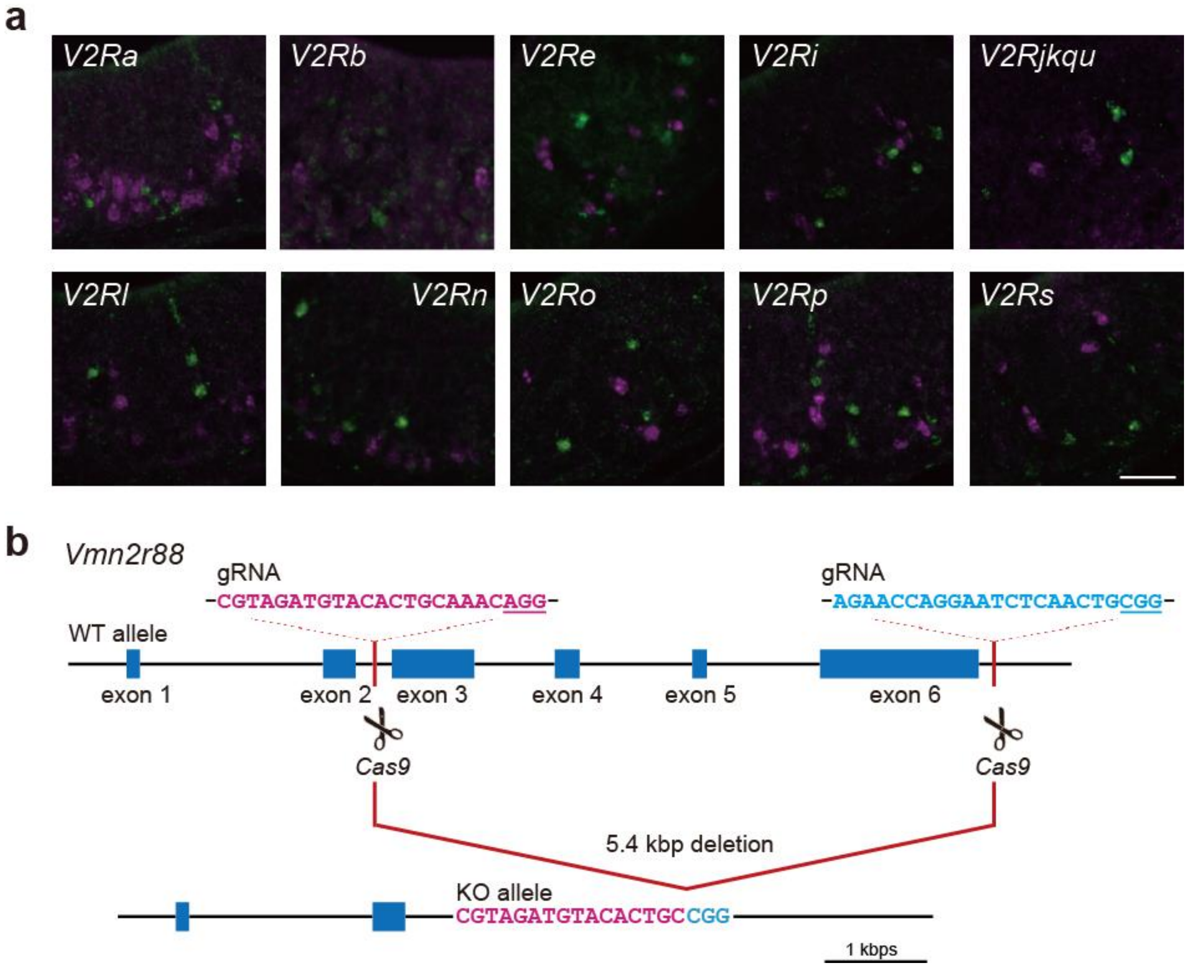

Supplementary Fig. 3 Representative images from hemoglobin specific receptor screening and schematic diagram illustrating the generation of *Vmn2r88*-deficient mice**. a** Representative images of a VNO section from a hemoglobin-stimulated mouse labeled by ISH with *Egr1* (green) and *V2R* clade-specific cRNA probes without the *V2Rf* clade (magenta). $n = 3$. Scale bar, 50 µm. **b** Two guide RNAs (shown in magenta and cyan) were designed to induce Cas9-mediated double strand breaks flanking exon 3-6, which encodes the entire transmembrane domain of V2R. The non-homologous end joining following the dual double strand breaks results in the entire deletion of exons 3-6. The junction sequence was confirmed by genomic PCR.

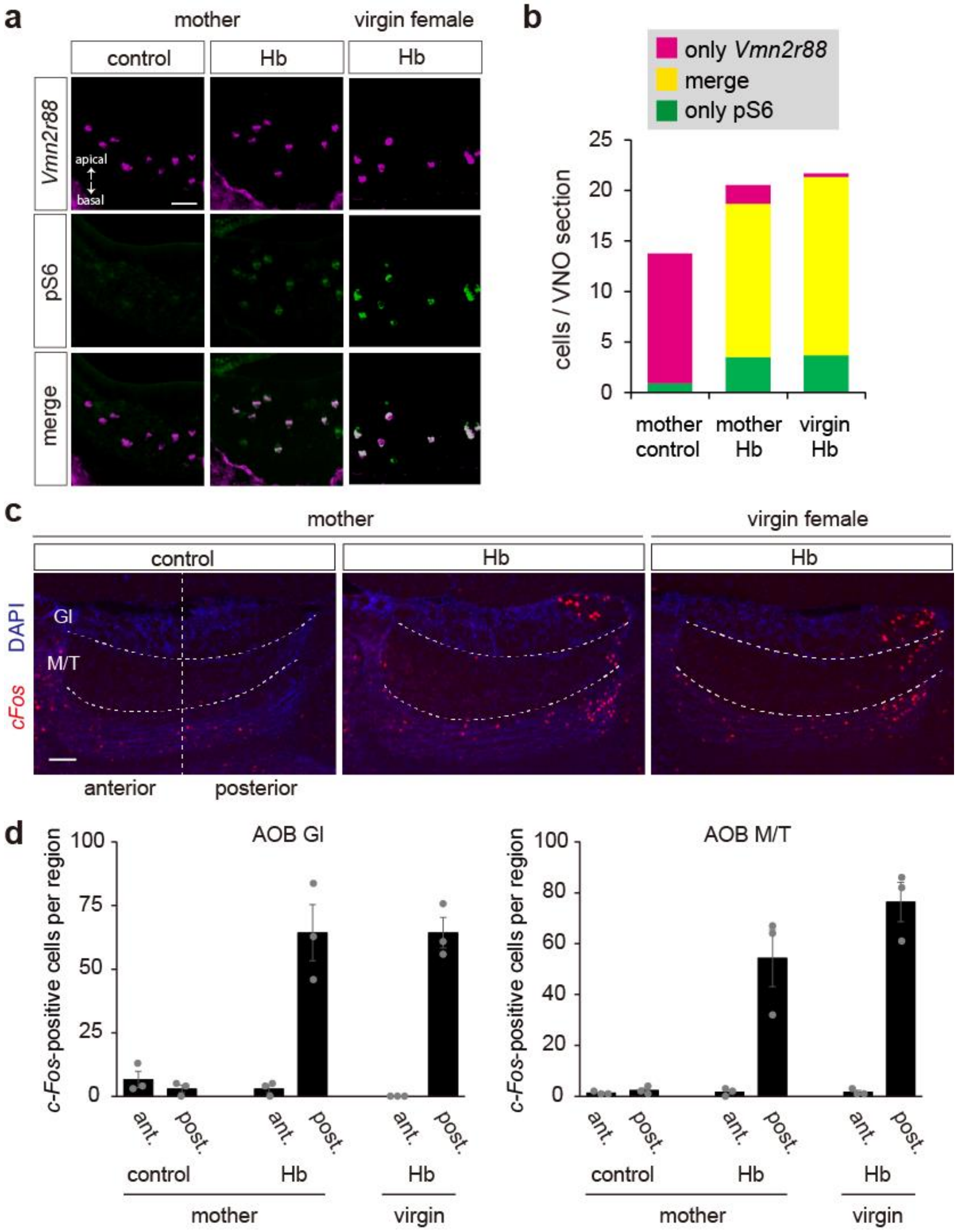

Supplementary Fig. 4 Hemoglobin activates Vmn2r88-expressing cells in the VNO and neurons in the AOB in both lactating and virgin females. **a** *Vmn2r88* ISH (magenta) and pS6 (green) immunostaining of VNO sections from mother or virgin female mice exposed to hemoglobin (Hb) or distilled water (control). $n = 3$ for virgin female, $n = 4$ for mother-control and mother-Hb. Scale bar, 50 µm. **b** Quantification of visualized neurons in the VNO. The number of pS6- (green), *Vmn2r88*- (magenta), and double-positive cells (yellow) per VNO section were counted. 9 sections from each of 3 animals were quantified. **c** Representative

immunohistochemical images (left: mother exposed to water, middle: mother exposed to Hb (300 μg), right: virgin female exposed to Hb) of ISH with *c-Fos* probes in the sections including the AOB. *n* = 3 for each condition (mother-control, mother-Hb, and virgin female-Hb). Scale bar, 100 μm. Abbreviations: Gl, glomerular layer; M/T, mitral/tufted cell layer. **d** Quantification of *c-Fos*-positive neurons in the glomerular layer (Gl) and mitral/tufted cell layer (M/T) of the AOB. The number of sections counted to determine the number of *c-Fos*-positive neurons in each animal was 6. Error bars, S.E.M. *n* = 3 for each condition. Abbreviations: ant, anterior; post, posterior.

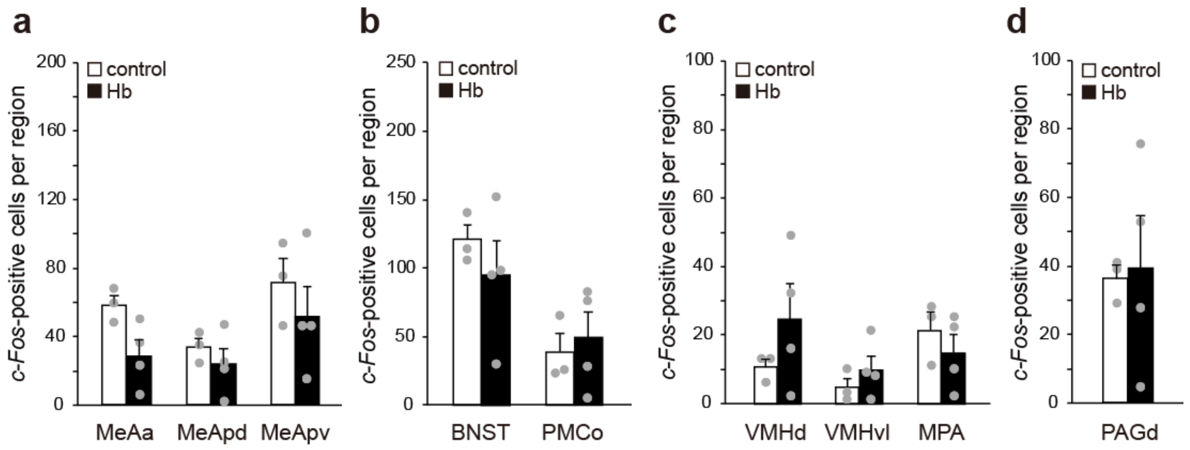

Supplementary Fig. 5 Histological analysis in higher brain regions with *Vmn2r88*-deficient lactating mothers**. a-d** The total number of *c-Fos*-positive cells in each sub-region of the MeA **(a),** BNST and PMCo **(b)**, VMH and MPA **(c)**, and PAGd **(d)** of *Vmn2r88*-deficient mothers stimulated with control buffer or hemoglobin (Hb). *n* = 3 for control, and *n* = 4 for Hb. 6 (MeAa), 8 (MeAp), 7 (BNST), 5 (PMCo), 10 (VMH), 4 (MPA) and 4 (PAG) sections from each animal were quantified. Error bars, S.E.M.

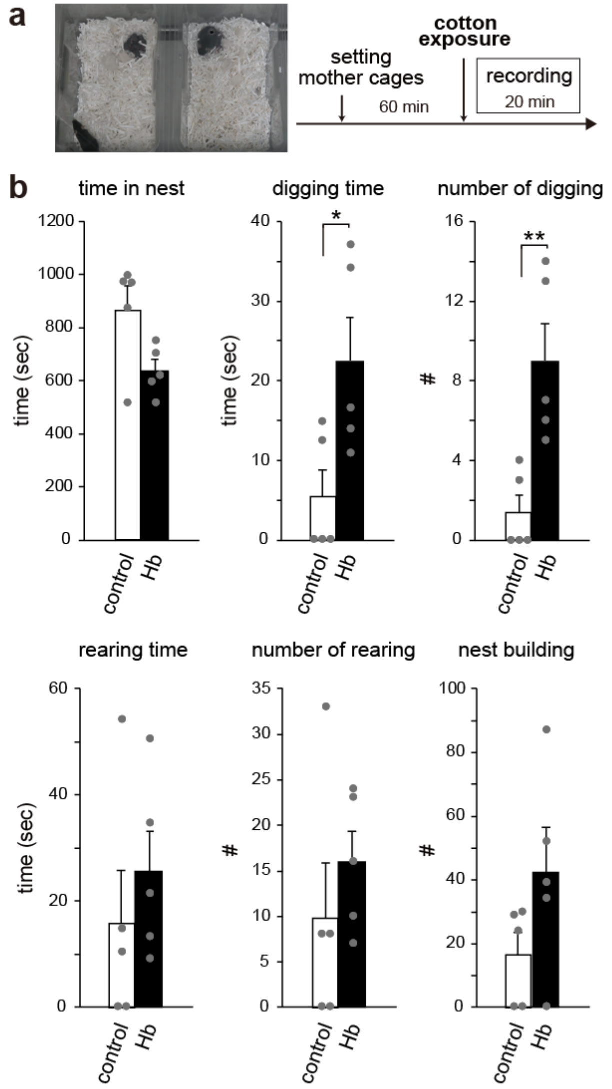

Supplementary Fig. 6 Hemoglobin enhances digging behavior of lactating females with pups**. a** Schematic image and illustration of the setup and timeline for digging behavior observation of C57BL/6 lactating mothers with their pups. **b** Quantification of the time in the nest, digging time, number of digging, rearing time, number of rearing, and number of nest buildings, of C57BL/6 lactating mothers pre-stimulated with control buffer- or hemoglobin (Hb)-cotton swabs during a 20-minute recording. n = 5. Error bars, S.E.M. (digging time) p=0.0284 and (number of digging) p=0.006 by unpaired two-sided Student's t-test. Hemoglobin-dependent enhancement in total duration and number of digging behavior was observed.

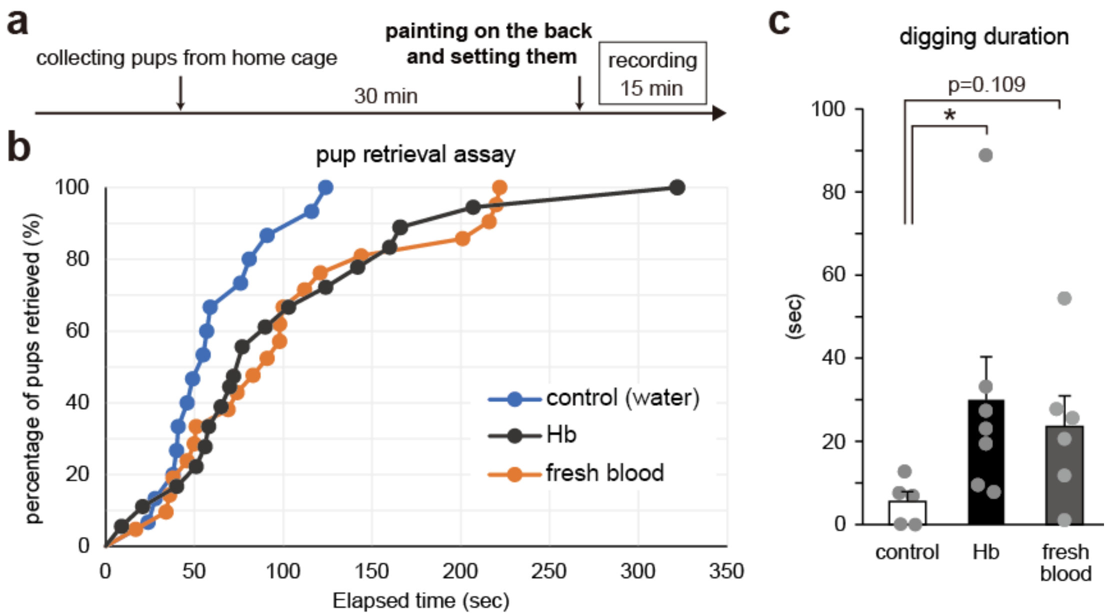

Supplementary Fig. 7 Pup retrieval assay with C57BL/6 lactating female mice**. a** Timeline for pup retrieval assay using lactating female mice and pups painted either hemoglobin (Hb, 300 µg), fresh blood (30 µL), or control buffer. Pups were removed from the female's cage 30 min prior to behavior recording. **b** Combined percentage of pups with Hb-, fresh blood-, or control buffer-painting on their back (Three pups were placed in each trial.) retrieved by an animal group as a function of time. **c** Quantification of the digging time duration of lactating mothers in pup retrieval assays. n = 5 for control, n =7 for Hb, and n =6 for fresh blood. Error bars, S.E.M. (control vs. fresh blood) p=0.109 and (control vs. Hb) p=0.031 by the two-sided Steel-Dwass test**.**

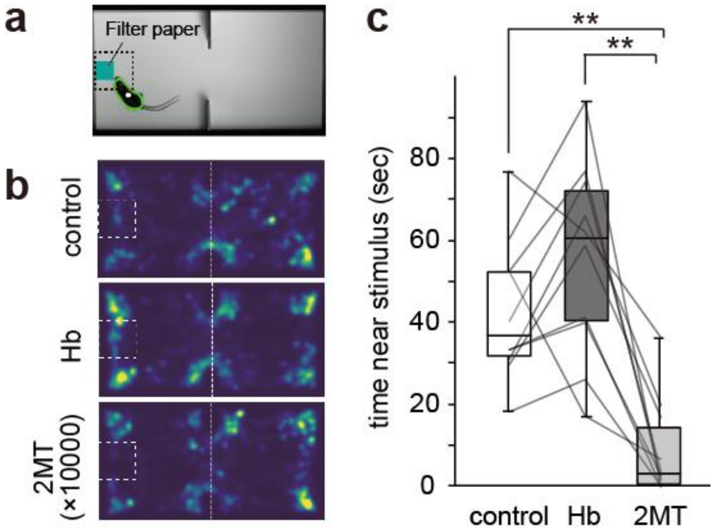

Supplementary Fig. 8 Hemoglobin does not possess negative valence**. a** Representative image of the two chamber assay with lactating female mice. After habituation, a piece of filter paper soaked with control buffer, hemoglobin (Hb), or 2MT (shown in cyan) was placed in one side of the chamber. The time spent near the filter paper (10 cm x 10 cm, framed with a dotted line) was quantified. **b** Heat maps showing the position of one representative animal. The ROI near the stimulus area is shown in a dashed white box. **c** Quantification of the time spent near the stimulus (10 x 10 cm square). *n* = 10. Error bars, S.E.M. The vaule of (maximun, 3rd quartile, mean, median, 1st quartile, minimum); (control: 76.7, 54.4, 42.8, 36.8, 30.7, 18.2), (Hb: 93.8, 75.0, 55.6, 60.3, 36.5, 17.0), (2MT: 36.2, 17.4, 8.7, 3.1, 0, 0). F=23.95, p=8.46 x 10^-6, (control vs. 2MT) p=0.0006, (Hb vs. 2MT) p=0.0016 by One-way ANOVA with repeated measures followed by Bonferroni's correction.

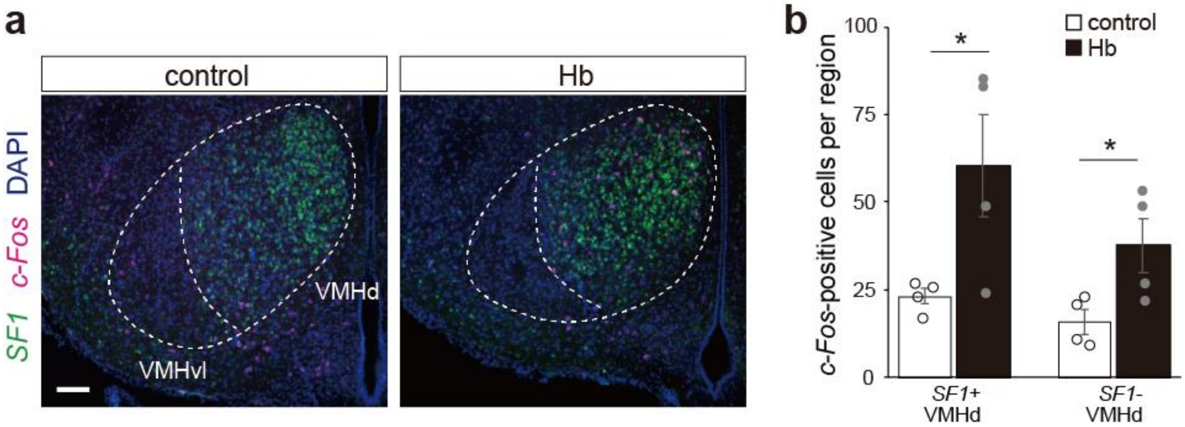

Supplementary Fig. 9 Hemoglobin activates SF1-expressing neurons, a specific molecule marker for the sub-region, in the VMHd. **a** Images from dual-color ISH staining of VNO sections from hemoglobin (Hb)- or distilled water (control)-stimulated C57BL/6 lactating female mice labeled with the *SF1* (green) and *c-Fos* cRNA probe (magenta). $n = 8$. Scale bar, 100 µm. **b** Quantification of *c-Fos*-positive neurons in the VMHd. The number of sections counted to determine the number of *c-Fos*-positive neurons in each brain area was 6. Error bars, S.E.M. $n = 4$ for control and Hb. (SF1+ VMHd) p=0.046 and (SF1- VMHd) p=0.043 by unpaired two-sided Student's *t*-test.

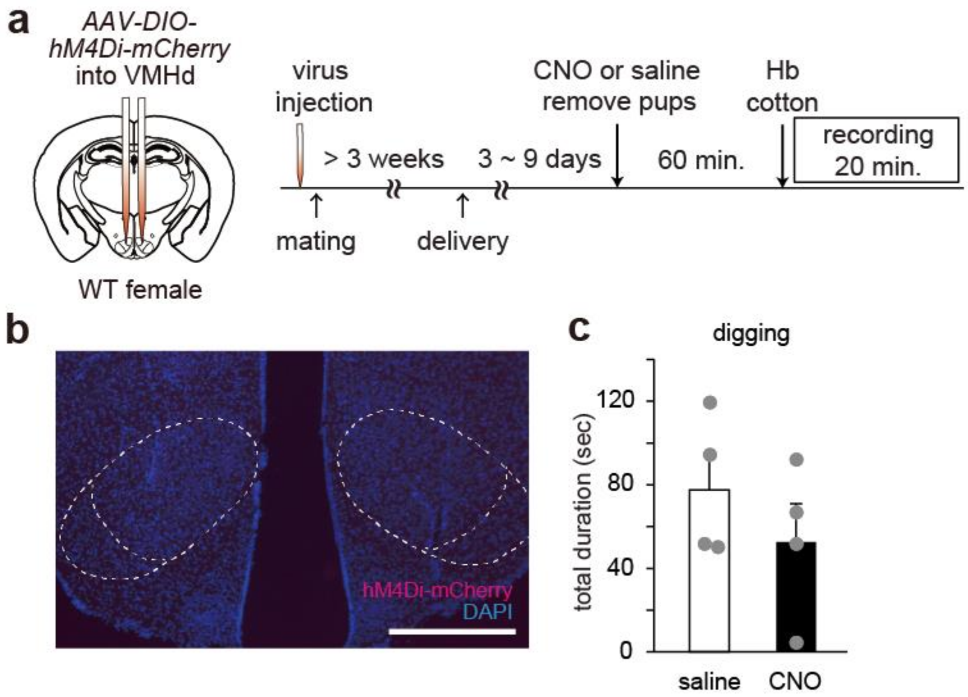

Supplementary Fig. 10 CNO administration itself does not induce decrease of digging behavior in wild type mother. **a** Schematic illustration of the animal setup and timeline for control of pharmacogenetic inhibition of *SF1* expressing neurons in the VMHd. *AAV-DIO-hM4Di-mCherry* is injected into the VMHd of wild type (WT) C57BL/6 female mice. Image adapted from Allen Mouse Brain Atlas[48]. **b** A representative coronal section checking DREADD-Gi expression (mCherry-positive cells shown in red if there is viral gene expression) in the VMHd. *n* = 8. Scale bar, 500 µm. **c** Quantification of the total digging duration (sec) of hemoglobin (Hb)-stimulated wild type C57BL/6 lactating mothers with pre-saline i.p. injection and CNO i.p. injection. *n* = 4 for saline and CNO. Error bars, S.E.M.

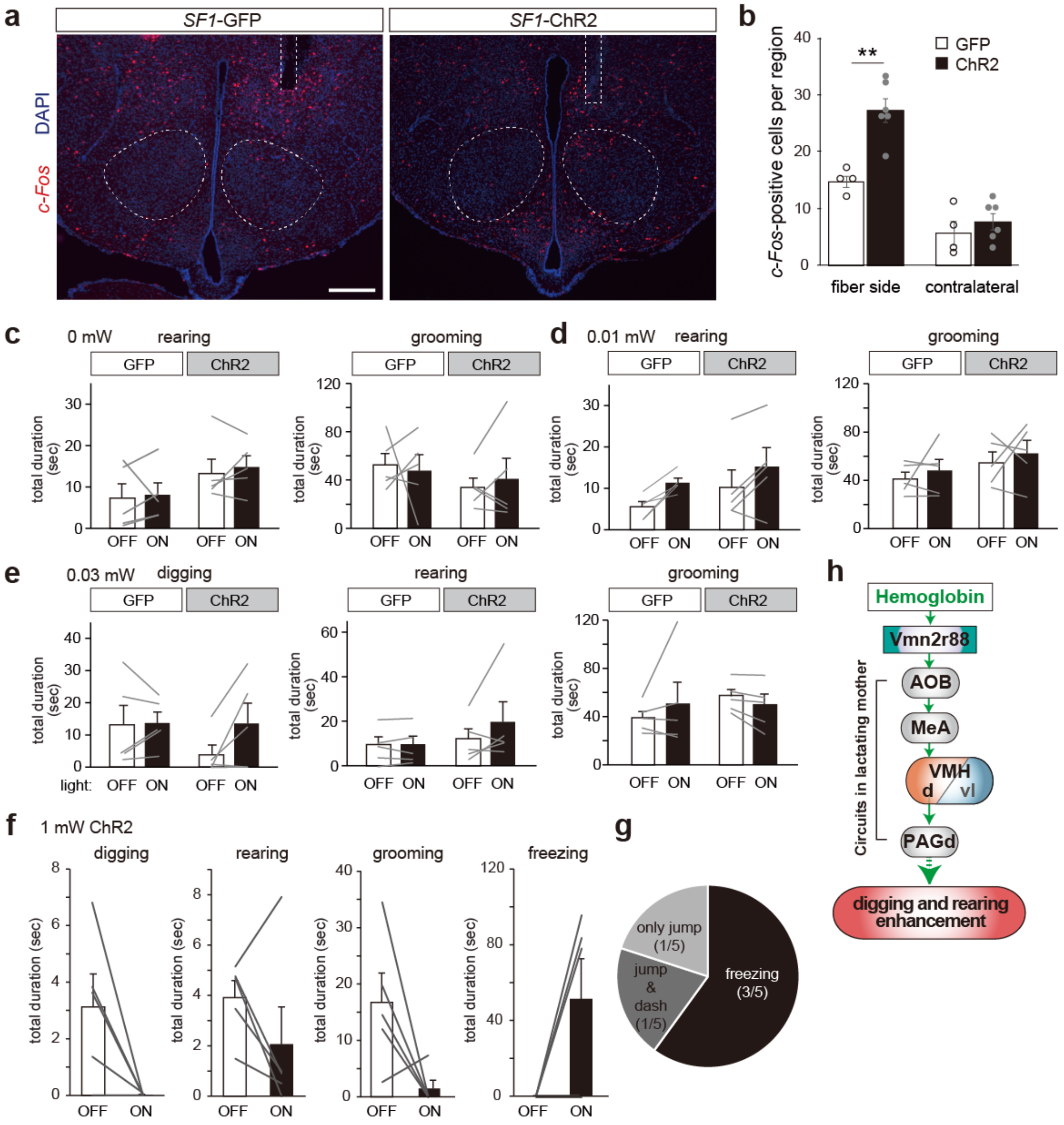

Supplementary Fig. 11 Activation of SF1-positive neurons in the VMHd elicits scalable behavior outputs**. a** Representative images of *c-Fos* ISH staining of brain sections from blue laser-stimulated (0.01 mW, 5 min) *SF1*-Cre mother mice injected with *AAV-DIO-GFP* (*SF1*-GFP, n = 4) or *AAV-DIO-ChR2* (*SF1*-ChR2, n = 6) in the VMHd. Scale bar, 100 μm. **b** Quantification of *c-Fos*-positive neurons in the VMHd from fiber trace containing sections. Error bars, S.E.M. p=0.001 by unpaired two-sided Student's *t*-test. **c-d** Quantification of

rearing, and self-grooming behaviors, with or without weak light stimulation (**c** 0 mW, **d** 0.01 mW). Error bars, S.E.M. *n* = 5. **e** Quantification of digging, rearing, and self-grooming behaviors, with or without weak light stimulation (0.03 mW). Error bars, S.E.M. *n* = 5. **f** Quantification of digging, rearing, self-grooming behaviors, and jumping, with or without stronger light stimulation (1 mW). Error bars, S.E.M. *n* = 5. **g** A pie chart showing the distribution of behavior outputs by lactating females stimulated by stronger light (1 mW). **h** Model of the neural pathways responsible for hemoglobin-mediated digging and rearing enhancement of lactating mothers.

References for Supplemental Information

1. Saitou, N. & Nei, M. The neighbor-joining method: a new method for reconstructing phylogenetic trees. *Mol. Biol. Evol.* **4,** 406–425 (1987).
2. Kumar, S., Stecher, G. & Tamura, K. MEGA7: Molecular Evolutionary Genetics Analysis Version 7.0 for Bigger Datasets. *Mol. Biol. Evol.* **33,** 1870–1874 (2016).
