# Supplementary material for: Hemoglobin in the blood acts as a chemosensory signal via the mouse vomeronasal system
Source: Nat Commun. 2022 Feb 3;13:556. doi: 10.1038/s41467-022-28118-w (PMC8814178; doi:10.1038/s41467-022-28118-w)
Supplement: Supplementary file 3 — Description of Additional Supplementary Files [file 41467_2022_28118_MOESM3_ESM.docx]

**Description of Additional Supplementary Files**

**Title: Supplementary Movie 1**

**Description:** A C57BL/6 lactating female mouse displays hemoglobin-mediated digging behavior (2.97 MB). Video showing the behavior of a C57BL/6 lactating female mouse after exposure to a hemoglobin-soaked cotton swab. The hemoglobin-stimulated lactating mother displays robust digging behavior.
