# Supplementary material for: Hemoglobin in the blood acts as a chemosensory signal via the mouse vomeronasal system
Source: Nat Commun. 2022 Feb 3;13:556. doi: 10.1038/s41467-022-28118-w (PMC8814178; doi:10.1038/s41467-022-28118-w)
Supplement: Supplementary file 5 — Reporting Summary [file 41467_2022_28118_MOESM5_ESM.pdf]

nature portfolio

Corresponding author(s): Kazushige Touhara
Last updated by author(s): Nov 25, 2021

Reporting Summary

Nature Portfolio wishes to improve the reproducibility of the work that we publish. This form provides structure for consistency and transparency in reporting. For further information on Nature Portfolio policies, see our Editorial Policies and the Editorial Policy Checklist.

Statistics

For all statistical analyses, confirm that the following items are present in the figure legend, table legend, main text, or Methods section.

n/a	Confirmed	
☐	☒	The exact sample size (n) for each experimental group/condition, given as a discrete number and unit of measurement
☐	☒	A statement on whether measurements were taken from distinct samples or whether the same sample was measured repeatedly
☐	☒	The statistical test(s) used AND whether they are one- or two-sided<br>*Only common tests should be described solely by name; describe more complex techniques in the Methods section.*
☒	☐	A description of all covariates tested
☐	☒	A description of any assumptions or corrections, such as tests of normality and adjustment for multiple comparisons
☐	☒	A full description of the statistical parameters including central tendency (e.g. means) or other basic estimates (e.g. regression coefficient) AND variation (e.g. standard deviation) or associated estimates of uncertainty (e.g. confidence intervals)
☐	☒	For null hypothesis testing, the test statistic (e.g. F, t, r) with confidence intervals, effect sizes, degrees of freedom and P value noted<br>*Give P values as exact values whenever suitable.*
☒	☐	For Bayesian analysis, information on the choice of priors and Markov chain Monte Carlo settings
☒	☐	For hierarchical and complex designs, identification of the appropriate level for tests and full reporting of outcomes
☒	☐	Estimates of effect sizes (e.g. Cohen's d, Pearson's r), indicating how they were calculated

Our web collection on statistics for biologists contains articles on many of the points above.

Software and code

Policy information about availability of computer code

Data collection	Light stimulation during optogenetics (Fig. 7d-g) is controlled by original code of Arduino IDE (https://link.springer.com/content/pdf/10.1007/s40799-021-00449-1.pdf).
Data analysis	Open field assay (Fig.6) was analyzed by ImageJ version 2.1.0 (https://www.nature.com/articles/nmeth.2089.pdf) with plugin named ImageOF (https://cbsn.neuroinf.jp/database/item/id/ImageOF). Tracking of two-chamber-test (Supplementary Fig. 8) was performed using original codes for python2. Spikes of electrophysiology (Fig.1i-j) were analyzed using Igor Pro functions (Wave Metrics, https://nyaspubs.onlinelibrary.wiley.com/doi/10.1111/j.1749-6632.2002.tb04544.x). Neighbor-joining phylogenic tree (Supplementary Fig. 2c) was constructed using MEGA7 (Kumar et al., Mol. Biol. Evol., 2016). Three dimensional structure of human hemoglobin (Fig. 2f) was analyzed by RCSB protein data bank (https://www.rcsb.org/structure/1a3n). R version 3.5.0 (https://www.r-project.org/) and ImageJ version 2.1.0 (https://www.nature.com/articles/nmeth.2089.pdf) were used for all non-parametric statistical analyses in this study.

For manuscripts utilizing custom algorithms or software that are central to the research but not yet described in published literature, software must be made available to editors and reviewers. We strongly encourage code deposition in a community repository (e.g. GitHub). See the Nature Portfolio guidelines for submitting code & software for further information.

Data

Policy information about availability of data

All manuscripts must include a data availability statement. This statement should provide the following information, where applicable:

- Accession codes, unique identifiers, or web links for publicly available datasets
- A description of any restrictions on data availability
- For clinical datasets or third party data, please ensure that the statement adheres to our policy

All of the data generated for this study are deposited into data source file and further information can be available from the corresponding author upon reasonable request.

Field-specific reporting

Please select the one below that is the best fit for your research. If you are not sure, read the appropriate sections before making your selection.

☒ Life sciences ☐ Behavioural & social sciences ☐ Ecological, evolutionary & environmental sciences

For a reference copy of the document with all sections, see nature.com/documents/nr-reporting-summary-flat.pdf

Life sciences study design

All studies must disclose on these points even when the disclosure is negative.

Sample size	Sample sizes were based on comparable n-values from the literature published previously (see ref.4,12,14,15)
Data exclusions	In the assay of optogenetics (Fig. 7d-g), we excluded the data of 3 mice whose optic fibers were not on the targeted region, the VMHd. In Fig. 5c, we excluded the result of one animal that widely separated from the average after performing Smirnov-Grubbs test. There is no data exclusion from the other assays.
Replication	All of the histological and electrophysiological analyses were replicated with at least three independent animals or biological samples. We show representative images and describe the exact n values in the corresponding legends. All of the behavioral experiments are replicated with at least three independent animals. The exact n values are described in the corresponding legends. Especially, Fig.5b, Fig. 6b, and Fig. S7 were replicated with multiple experimenters. Optogenetic assay in Fig. 7d-g was done by KKI initially with male mice and the data was replaced by TI with lactating female mice during the review.
Randomization	All the mice were randomly assigned to each experimental groups in all the experiments in the study.
Blinding	There was the process of blinding when analyzing all the data in this study. During the process of human annotation or cell counting for the all experiments before the initial submission of the manuscript, the name of video or sample was hidden from experimenters to perform blinding. The human annotation for behavioral assay during the process of review was performed by person who had not performed assays.

Reporting for specific materials, systems and methods

We require information from authors about some types of materials, experimental systems and methods used in many studies. Here, indicate whether each material, system or method listed is relevant to your study. If you are not sure if a list item applies to your research, read the appropriate section before selecting a response.

Materials & experimental systems

n/a	Involved in the study	
☐	☒	Antibodies
☒	☐	Eukaryotic cell lines
☒	☐	Palaeontology and archaeology
☐	☒	Animals and other organisms
☒	☐	Human research participants
☒	☐	Clinical data
☒	☐	Dual use research of concern

Methods

n/a	Involved in the study	
☒	☐	ChIP-seq
☒	☐	Flow cytometry
☒	☐	MRI-based neuroimaging

Antibodies

Antibodies used	anti-c-Fos antibody (Oncogene (Ab-2), (1:1000) (lot# 21584-1), Merck (Ab-5), (1:100) (lot# ab7963-1), and Millipore (Calbiochem) (Ab-5), (1:10000) (lot# 34095)<br>biotinylated goat anti-rabbit IgG secondary antibody (1:200, Vector Laboratories)<br>anti-pS6 ribosomal protein (S235/236) antibody (1:200) (Cell Signaling, #4858) and anti-Gαo antibody (1:500) (MBL, #551)

	Alexa Fluor 488-conjugated goat anti-rabbit secondary antibody (1:500) (Invitrogen, A11034)<br>anti-Vmn2r88 antibody (1:2000) (originally made in this study)<br>Alexa Fluor 488-conjugated goat anti-guinea pig IgG secondary antibody (1:500) (Invitrogen, A11073) and Cyanine3-conjugated goat anti-rabbit IgG secondary antibody (1:500) (Invitrogen, A10522)<br>HRP conjugated anti-FITC antibody (1:250) (PerkinElmer, #NEF710001EA)<br>HRP conjugated anti-DIG antibody (1:250) (Roche Applied Science, #11207733910)
Validation	All antibodies except anti-Vmn2r88 antibody made in this study have been validated by the suppliers with relevant images on their websites. Additionally, all antibodies have been cited multiple times as listed on the manufacture's website except discontinued cFos antibodies that we could not check anymore. Specifically, for the primary antibodies used in this paper,<br><br>anti-c-Fos antibody (Oncogene (Ab-2)) relevant citation: Wersinger SR and Maun MJ (Journal of Neuroendocrinol, 1996), Kimoto et al (Nature, 2005)<br>anti-c-Fos antibody (Merck (Ab-5)): Shirasu et al (Neuron, 2014)<br>anti-c-Fos antibody (Calbiochem (Ab-5)): Tsunoda et al (Current Biology, 2018)<br>anti-pS6 ribosomal protein (S235/236) antibody (Cell Signaling, #4858): Osakada et al (Nature Communications, 2018), https://www.cellsignal.com/products/primary-antibodies/phospho-s6-ribosomal-protein-ser235-236-d57-2-2e-xp-rabbit-mab/4858<br>anti-Gαo antibody (MBL, #551): Kimoto et al (Nature, 2005), https://www.mblbio.com/bio/g/dtl/A/?pcd=551<br>anti-Vmn2r88 antibody (originally made in this study): Validated by staining with sections of the VNO from Vmn2r88-knock out mice (Fig. 3d-e)<br>HRP conjugated anti-FITC antibody (PerkinElmer, #NEF710001EA): Ishii et al (Neuron, 2017), https://www.perkinelmer.com/product/antifluorescein-hrp-nef710001ea<br>HRP conjugated anti-DIG antibody (Roche Applied Science, #11207733910): Ishii et al (Neuron, 2017), https://www.sigmaaldrich.com/US/en/product/roche/11207733910

Animals and other organisms

Policy information about studies involving animals; ARRIVE guidelines recommended for reporting animal research

Laboratory animals	Animals were housed under a regular 12 h dark/light cycle, 23 ± 2 °C, 50 % humidity with food and water ad libitum. Wild type BALB/c, C57BL/6 were purchased from Japan CLEA (Japan), Japan SLC (Japan) or Charles River Japan (Japan) for all experiments. Vmn2r88-deficient mice were generated as described in the method section of "Generation of mutant mice by CRISPR-mediated genome editing". SF1-Cre (also known as Nr5a1-Cre, Jax#012462) mice were purchased from the Jackson Laboratory. Animals used for blood collection and purification of hemoglobin are 10-12-weeks-old BALB/c and C57BL/6 male. Animals for electrophysiology were 10-week-old BALB/c female mice. All animals when exploring vomeronasal ligand and its receptor (Fig.1, 2, 3 and Supplementary Fig. 1, 2, 3) were 10-12-weeks-old BALB/c female mice and the other staining experiments to figure out brain regions activated by hemoglobin including originally generated Vmn2r88-deficient mice were 10-12-weeks-old C57BL/6 male, female, and 5-7-month-old lactating female mice (Fig. 4 and Supplementary Fig. 4,5). In the behavior assays with lactation experienced female mothers including SF1-Cre mice for neural manipulation, we used 5-10 months-old C57BL/6 lactating female mice after 2-3 times of lactation experiences. In the experiments shown in Fig. 5d, 10-12-weeks-old C57BL/6 male and virgin female mice were used.
Wild animals	The study did not involve wild animals.
Field-collected samples	The study did not involve field samples.
Ethics oversight	Experiments were carried out in accordance with the animal protocols approved by the Animal Care and Use Committees at the University of Tokyo and RIKEN.

Note that full information on the approval of the study protocol must also be provided in the manuscript.
